# A Comprehensive Review of Vision-Based 3D Reconstruction Methods

**DOI:** 10.3390/s24072314

**Published:** 2024-04-05

**Authors:** Linglong Zhou, Guoxin Wu, Yunbo Zuo, Xuanyu Chen, Hongle Hu

**Affiliations:** Key Laboratory of Modern Measurement and Control Technology Ministry of Education, Beijing Information Science and Technology University, Beijing 100080, China; linglongzhou@bistu.edu.cn (L.Z.); zuoyunbo@126.com (Y.Z.); xuanyuc2023@163.com (X.C.); ershisan@163.com (H.H.)

**Keywords:** static 3D reconstruction, dynamic 3D reconstruction, 3DGS, deep learning, NeRF

## Abstract

With the rapid development of 3D reconstruction, especially the emergence of algorithms such as NeRF and 3DGS, 3D reconstruction has become a popular research topic in recent years. 3D reconstruction technology provides crucial support for training extensive computer vision models and advancing the development of general artificial intelligence. With the development of deep learning and GPU technology, the demand for high-precision and high-efficiency 3D reconstruction information is increasing, especially in the fields of unmanned systems, human-computer interaction, virtual reality, and medicine. The rapid development of 3D reconstruction is becoming inevitable. This survey categorizes the various methods and technologies used in 3D reconstruction. It explores and classifies them based on three aspects: traditional static, dynamic, and machine learning. Furthermore, it compares and discusses these methods. At the end of the survey, which includes a detailed analysis of the trends and challenges in 3D reconstruction development, we aim to provide a comprehensive introduction for individuals who are currently engaged in or planning to conduct research on 3D reconstruction. Our goal is to help them gain a comprehensive understanding of the relevant knowledge related to 3D reconstruction.

## 1. Introduction

Vision, serving as one of the most important capabilities of human beings, enables humans to recognize and interact with the 3D physical world. The digital representation and construction of 3D scenes in computers form the foundation for many crucial applications today. This is evident from the increasing number of relevant papers published in well-known international conferences and journals, showcasing the degree of research development in this field. In many cases, three-dimensional reconstruction technology provides an alternative to replicating real objects for precious or delicate cultural heritage artifacts, avoiding the overly invasiveness that traditional plaster casting techniques may bring. It is also useful for protecting historical relics, cultural heritage, etc. plays an extremely important role [1]. In the game and movie industry, dynamic 3D scene reconstruction can be used for real-time rendering to enhance the viewing experience of games and movies [2]. In medical imaging, it is used to construct patient-specific organ models for surgical planning [3,4,5]. In robot navigation, dynamic 3D scene reconstruction enables the robot to better comprehend its surrounding environment and improve navigation accuracy and safety [6]. In the field of industrial design, 3D reconstruction technology can assist in creating precise digital models by capturing the 3D geometric information of real objects. It aids users in comprehending the dynamic changes in data [7]. By capturing the user’s body shape, needs, or preferences, designers can personalize customized products [8]. Additionally, it can capture and record the geometry and structure of actual equipment or mechanical parts, providing a digital foundation for equipment maintenance [9].

Different from traditional manual 3D modeling using Computer-Aided Design (CAD) software or Digital Content Creation (DCC) software, 3D reconstruction technology aims to begin with sensor input, such as pictures, point clouds, and other data. The corresponding 3D structure and scene are automatically reconstructed without manual intervention. In the field of photogrammetry, German scientists Albrecht Meydenbauer and Carl Pulfrich made significant contributions to image measurement and photogrammetry from the late 19th to the early 20th century. Their work provided a theoretical basis for subsequent 3D measurement and reconstruction [10]. The photogrammetry method mainly captures images through cameras and then uses image processing and measurement techniques to obtain the 3D information of the target object. In the 1960s, MIT’s Roberts used computer programs to extract 3D structures of polyhedra, such as cubes, wedges, and prisms, from digital images. After studying multiple visual images of the building block world system, he believed that in the building block world, 3D objects can be simply represented by two-dimensional shape combinations [11]. This pioneering research laid the foundation for studying 3D reconstruction to understand 3D scenes. In the 1970s, Marr and Poggio of the Massachusetts Institute of Technology proposed a theoretical framework for how the visual system reconstructs 3D structures from two-dimensional images [12]. The core of this theory is to eliminate false matches, integrate previous advances in stereo matching, and posit that the difficulty of stereo matching is related to image parallax. The range is proportional to the resolution. Shortly after Marr proposed this theory, Grimson [13] further implemented the algorithm and demonstrated its applicability to natural image matching.

3D reconstruction is divided into explicit and implicit expression methods based on different approaches, offering diverse perspectives and processing techniques for data obtained from the real world. Explicit expression refers to a representation method that clearly defines geometric shapes and structures to directly describe the external or internal geometry of an object. It is a discretized expression, which inevitably leads to a loss of information, necessitating the development of new processes. There is a significant overhead when synthesizing images from different perspectives. Implicit expression describes the geometry of an object through a function instead of directly providing its geometric representation. In implicit representation, the geometry of the object is implicitly defined by an implicit function or implicit surface equation, and the function is used to solve the problem. Values can be obtained from points on the surface.

### 1.1. Explicit Expression

The main methods for displaying data include point clouds, voxels, and meshes. Point clouds consist of discrete data collected from various sensors or scanning devices. It is used to represent the external surface of an object or the spatial structure of a scene. A point cloud is an unordered collection of points in 3D space. Divide the 3D space into uniform cubic units. Each cubic unit is called a voxel. Each voxel can contain information representing spatial attributes, such as color, density, or depth. Voxels are commonly utilized in medical image processing, computational fluid dynamics, and other fields. Voxel storage is used to represent the structure and attributes within a space, but it has high space complexity. The mesh is composed of connected vertices, edges, and faces. The mesh model can be composed of triangles, quadrilaterals, or higher-order polygons, and can describe most topological structures. It can accurately represent complex geometric shapes and details. The surface described by each triangle is planar, making it suitable for numerous computer graphics and engineering applications where triangle meshes are commonly used. This ensures that the projection is always convex and easy to rasterize.

### 1.2. Implicit Expression

Implicit expression does not require explicit storage of geometric data; so, it offers advantages in saving storage space and processing complex geometries. However, computing the value of an implicit function can be time-consuming, and understanding and manipulating the implicit expression can be challenging. Implicitly represented 3D models can be determined by continuous decision boundaries, enabling shape recovery at any resolution. Commonly used implicit representations include implicit surfaces, Signed Distance Function (SDF), Occupancy Field, Radiance Field, etc.

Implicit surfaces can be composed of equations of curves or surfaces, such as Bézier curves, Bézier surfaces, NURBS, etc. Implicit surfaces can offer more precise and adaptable representations, making them suitable for scenarios where accurately modeling and designing simple geometric shapes is essential. For complex geometric structures, intricate mathematical descriptions are necessary. In SDF, the value of each point represents the signed distance from the point to the nearest object surface. This distance can be a positive value (indicating that the point is outside the object), a zero value (indicating that the point is on the object’s surface), or a negative value (indicating that the point is on the surface of the object). The form of SDF can usually be expressed as D(p), where p represents the point coordinates in 3D space. SDF implicitly represents the geometry of an object through a function instead of directly providing the geometry of the object. The occupancy field is a mapping from one vector to another vector or a number. The field in space can be considered as the mapping from a “space midpoint” to “point attributes”; in other words, each point corresponds to the attributes of that point. The radiation field maps a point in space, a ray emitted by the point to the density value of the point, and the color value corresponding to the direction of the ray [14].

In practical applications, explicit and implicit expressions are often used in combination [15]. The comprehensive utilization of the advantages of both explicit and implicit expressions can enhance the modeling, analysis, and processing of 3D models. Explicit expressions offer advantages in intuitiveness and accuracy, while implicit expressions have unique features in flexibility and storage efficiency. Choosing the appropriate expression or comprehensive application based on specific application requirements is crucial. It is an impossible task to cover all possible 3D reconstruction techniques in this survey; so, we have chosen representative techniques among them.

The remainder of this paper is structured as follows: Section 2 provides an overview of static 3D reconstruction methods, details traditional static 3D reconstruction methods, and includes computer graphics techniques related to 3D reconstruction. Section 3 summarizes and discusses dynamic 3D reconstruction methods, including the currently popular 3DGS. Section 4 introduces 3D reconstruction based on machine learning in detail, focusing on the application of deep learning technology in 3D reconstruction. Section 5 introduces commonly used datasets for 3D reconstruction, including human bodies and indoor and outdoor scenes. Section 6 discusses the application prospects and challenges of 3D reconstruction technology. Finally, Section 7 summarizes the work presented in this paper.

## 2. Traditional Static 3D Reconstruction Methods

Most creatures in nature, including humans, rely on vision to perceive and reconstruct 3D objects in the physical world. 3D reconstruction can be categorized into sparse reconstruction and dense reconstruction based on the density of information acquired. Sparse reconstruction focuses on obtaining the accurate 3D positions of a small number of key points or feature points in the scene. It utilizes techniques such as feature point matching and key point extraction to represent the geometric shape of the entire scene through these discrete points. Dense reconstruction aims to obtain the accurate 3D coordinates of each pixel in the scene. By estimating the depth of each pixel in the image, the system generates a dense depth map, point cloud, or voxel, enabling high-density reconstruction of the entire scene. Develop a model to create a comprehensive description of the entire scene.

In 1997, Varady et al. categorized data acquisition methods into two types: contact and non-contact [16]. The contact method uses specific instruments to quickly and directly measure the 3D information of the scene [17], which mainly includes trigger measurement and continuous measurement. The contact method can only be used in situations where the instrument can come into contact with the measurement scene, such as coordinate measuring machines (CMMs), etc. The non-contact method utilizes image analysis models to acquire data from the measured object without physically touching it. The non-contact 3D reconstruction process involves capturing an image sequence using visual sensors (one or more cameras). Subsequently, relevant information is extracted, and finally, reverse engineering modeling is conducted using this information to reconstruct the 3D structural model of the object [18]. In 2005, Isgro et al. [19] divided non-contact methods into two categories: active and passive.

### 2.1. Active 3D Reconstruction Methods

Active methods of vision-based 3D reconstruction involve mechanical or radiometric interference with the reconstructed object to acquire depth maps. These methods include structured light, laser rangefinders, and other active sensing technologies. Among them, 3D reconstruction technologies based on active methods mainly include the laser scanning method [20,21], industrial computed tomography (CT) scanning [22], structured light method [23], time-of-flight (TOF) technology [24], shadow method [25], etc. These methods primarily utilize optical instruments to scan the surface of an object and reconstruct the 3D structure by analyzing the scanned data.

#### 2.1.1. Laser Scanning

**Figure 1:**
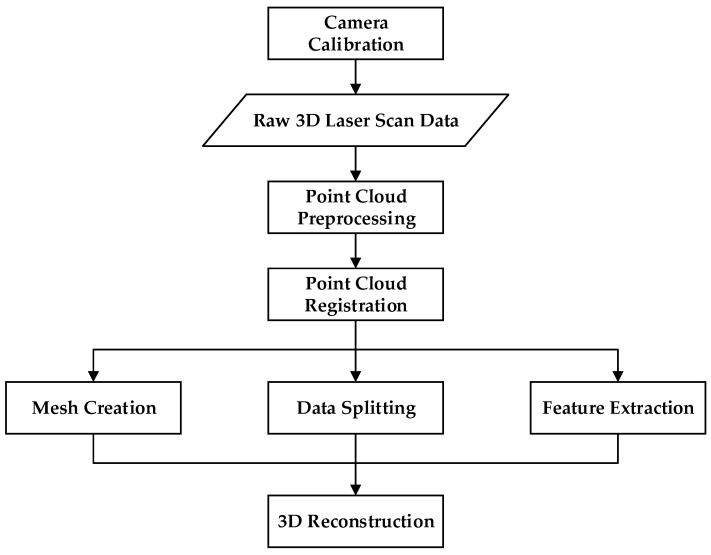
3D reconstruction process based on laser scanning.

The system scans the target surface with a laser beam emitted by a laser scanner and LiDAR [26]. It combines the controlled steering of the laser beam with a laser rangefinder, measures the reflection or scattering of the laser, and calculates the distance on the object’s surface. This method enables the rapid capture of the surface shape of objects, buildings, and landscapes by conducting distance measurements in all directions [27,28]. A 3D model created by laser scanning data collected with 3D laser scanning technology is represented as a point cloud. 3D laser scanning can rapidly capture millions of point clouds, providing an accurate representation of the characteristics of the measured target surface. It has the characteristics of high precision and high density, providing a guarantee for 3D modeling and visualization. However, it is not suitable for transparency and reflection. Surface objects are less efficient. The process of 3D reconstruction using laser scanning method is shown in Figure 1.

In 1999, Yang et al. [29] proposed triangulation laser scanning, in which the laser point, camera, and laser emitter form a triangle. They discussed in detail the factors that affect the accuracy of laser scanning measurements based on the principle of large-scale curved surface measurements [30]. Boehler et al. [31] analyzed and verified the impact of using different types of 3D laser scanners on experimental results. Voisin et al. [32] studied the impact of ambient light on 3D laser scanning. Tachella et al. proposed using coded aperture [33] to compress data by considering a subset of wavelengths for each pixel to achieve real-time 3D reconstruction. The laser line [34] reflected from the front surface of the target is used to enhance the accuracy of stereoscopic vision reconstruction of transparent or translucent objects.

The laser scanning method is primarily used in terrain surveying, architectural scanning, cultural relic protection, manufacturing, virtual reality, and other fields. Among them, the laser scanning method is a fundamental component of Building Information Modeling [35]. It can create 3D records and archives of engineering construction, providing a real data foundation for subsequent maintenance. The articulated arms of modern coordinate measuring machines and robots are equipped with non-contact laser scanners. 3D holographic projection technology [36] uses the principle of holographic imaging to present optical information in a 3D form and generate realistic 3D images in space, allowing observers to experience real 3D visuals.

#### 2.1.2. CT Scanning

A CT scan is typically an X-ray computed tomography scan, which utilizes radiation to produce a 3D internal and external representation of the scanned object [37,38]. Some of the primary applications of CT scanning include defect detection, failure analysis, metrology, assembly analysis, and reverse engineering applications [39].

In 1972, Godfrey Hounsfield invented the CT scanner for medical imaging, thereby introducing CT scanning technology. Many advancements in CT scanning have allowed it to be used for metrology in the industrial field [40], in addition to its primary application for visual inspection in the medical field (medical CT scanning). Lorensen et al. proposed the Marching Cubes algorithm [41] and outlined the fundamental process of 3D surface reconstruction of medical images. Evans et al. [42] directly converted the 3D image data from X-ray computed tomography into a grid to model complex geometries, such as composite materials, or to accurately represent precision components at the microscopic scale. Uhm et al. aggregated reconstructed 3D models from multiple CT phases by aligning multiphase CT images [43] to generate a fused model with well-defined surfaces.

#### 2.1.3. Structured Light

**Figure 2:**
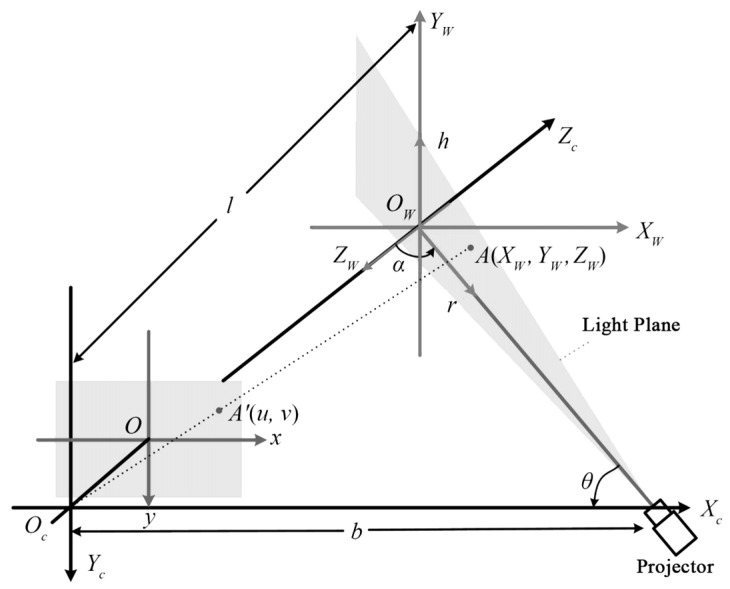
Principle diagram of structured light triangulation [18].

The structured light method involves projecting a specific pattern of light onto the scene and using a camera to capture the shape and deformation of the light spot. This process helps to infer the 3D structure of the object’s surface. The principle of structured light triangulation is shown in Figure 2. In 2000, Kowarschik et al. [44] utilized a 3D measurement system based on the grating structure method to address the occlusion issue of structured light in measurements. In order to ensure the quality of the light strip image, Zhang et al. [45] obtained multiple light strips by controlling the exposure time, took pictures, and then fused the pictures to create a light stripe image with enhanced quality. Ekstrand et al. [46] estimated the exposure time by analyzing the object’s surface, resulting in an improved light stripe image. Yang et al. [47] achieved a better light stripe image by adding controllable Liquid Crystal on Silicon (LCoS) for imaging with a wider dynamic range of the camera. Jiang et al. [48] utilized a cubic polynomial curve to fit the center point of the line structure light obtained through the weighted gray center of gravity method. This approach yielded smooth pixel coordinates of the light bar’s centerline, enhancing the accuracy of center extraction. The structured light method has strong real-time performance in 3D reconstruction [9], but its effectiveness is limited in environments with insufficient or excessive brightness. Santolaria et al. [49] integrated the line-structured light sensor and the articulated arm measurement system and provided a method for system integration. The use of mechanical projectors [50] improves the real-time performance of 3D reconstruction. Liu et al. utilized a rapid rotating mechanical projector (RMP) [51], which can be obtained with a shorter camera exposure time through the error diffusion binary encoding method and chrome plating technology. High-quality projected fringes, while introducing a probability distribution function algorithm to correct errors, ensuring the accuracy of the corresponding 3D shape measurement system. Zhang et al. utilized a white plane calibration target matrix [52] to streamline the parallel-axis structured light system and enhance the accuracy of the 3D reconstruction model.

#### 2.1.4. TOF

TOF technology continuously emits light pulses (typically invisible light) towards the object under observation and then employs a sensor to detect the light reflected back from the object. It determines the target distance by measuring the flight (round trip) time of the light pulse. It is commonly used in cameras and lasers. The TOF method is divided into Pulsed Modulation and Continuous Wave Modulation based on different modulation techniques. It is commonly used in outdoor 3D scanning, virtual reality, autonomous driving, and human posture detection.

Stipes et al. [53] utilized the Iterative Closest Point (ICP) algorithm to align the data from two TOF frames and executed the iterative process of ICP through the acquired 3D point cloud. Chua et al. [54] calculated the noise-weighted average range of the signal detection threshold and system noise to mitigate the impact of noise, demonstrating improved accuracy in distance reconstruction.

The 3D reconstruction method based on TOF has excellent real-time performance and is well-suited for complex environments. The current consumer-grade TOF depth cameras include Microsoft’s Kinect v2 in Redmond, WA, USA, MESA’s SR4000 in Zurich, Switzerland, Google Project Tango’s PMD Tech in San Jose, CA, USA, etc. These products have already been used in somatosensory recognition and gesture recognition. Environment modeling and other aspects have found numerous applications, with one of the most typical examples being Kinect v2.

#### 2.1.5. Photometric Stereo

It utilizes variations in illumination angles from multiple light sources to deduce the surface’s normal and depth by analyzing the changes in brightness on the object. It is suitable for objects with complex topological structures but is sensitive to lighting conditions. Woodham originally proposed Photometric Stereo in 1980 [55], and the special case where the data are a single image is called “shadow shape”, which was compared and analyzed by BKP Horn in 1989 [56]. For purely textureless objects with unknown surface reflectivity, especially non-Lambertian objects [57], use low-rank/RANSAC outlier rejection [58,59], factorization [60], and other methods. Karami et al. [61] utilized photogrammetry to produce geometric information and then combined it with the high spatial resolution of photometric stereo to obtain surface depth information. Ju et al. applied dual-position threshold normalization preprocessing to process the spatially varying reflectivity of non-Lambertian surfaces and adopted a parallel multi-scale feature extractor to preserve high-resolution representation and extract depth features [62]. 

Shadow photogrammetry utilizes light sources and cameras to deduce the shape and contour of an object by analyzing the shadow cast on its surface [63]. It involves capturing a series of images from a consistent viewpoint of a light source with a known movement pattern. Utilize the motion of cast shadows to reconstruct scene structure [64], especially effective for topologically simple objects [65,66].

#### 2.1.6. Multi-Sensor Fusion

Multi-source heterogeneous information fusion (MSHIF) comprehensively utilizes information obtained from different sensors, such as radar [67], lidar, camera, ultrasound, infrared thermal imager [68], GPS [69], MRI [70], IMU, and V2X, to overcome the limitations of individual sensors and create a more comprehensive perception of the environment or target, thereby enhancing the accuracy of 3D reconstruction [71]. Yu proposed a multi-modal 3D object reconstruction method based on variational autoencoders [72]. This method automatically determines the modality during training, which includes specific categories of information. It utilizes the transmission elements of the prior distribution to determine the pattern of latent variables in the latent space, enabling robust implementation of latent vector retrieval and 3D shape reconstruction.

### 2.2. Passive 3D Reconstruction Methods

The passive 3D reconstruction method based on vision does not interfere with the reconstructed object. It only uses optical sensors to measure the radiance reflected or emitted by the object’s surface and infers its 3D structure through the image [73].

#### 2.2.1. Texture Mapping

For objects with obvious texture features, utilizing the texture information on the object surface [74] to map the two-dimensional image to the 3D model can significantly enhance the realism of the model’s appearance. However, this process necessitates higher texture quality [75]. Lee et al. [76] directly associated the vertices of the implicit geometry with a voxel grid having texture coordinates and applied spatially varying perspective mapping to the input image, enabling real-time texture distortion and geometry update. Xu et al. [77] utilized background noise smoothing technology within a self-supervised framework to accomplish high-fidelity texture generation in high-resolution scenarios.

#### 2.2.2. Shape from Focus

The focusing method utilizes the camera’s focal length adjustment to calculate depth information by observing changes in the focal depth of the object. This is determined by the degree of image blur of the object at various focal lengths. Use a camera to capture images of the same scene at various focal lengths. In the image, the farther the object is from the focal plane, the blurrier its image will become. Depth estimation is another important aspect to consider. By utilizing the relationship between image blur level and depth, it is possible to estimate the object. The depth value of each part, and finally, the 3D reconstruction, convert the depth information into 3D coordinates, thereby obtaining the 3D reconstruction model of the object [78,79,80,81,82]. Yan et al. [83] used the multi-directional modified Laplacian operator to map the depth maps corresponding to different focal points and employed an iterative edge repair method to refine the reconstruction results. The focus method has better effects on objects with rich textures and does not require the use of multiple cameras or perspectives. However, it is more sensitive to lighting conditions. The texture method is often used for close-range shooting and is useful when dealing with low-texture or transparent objects. 

#### 2.2.3. Binocular Stereo Vision

**Figure 3:**
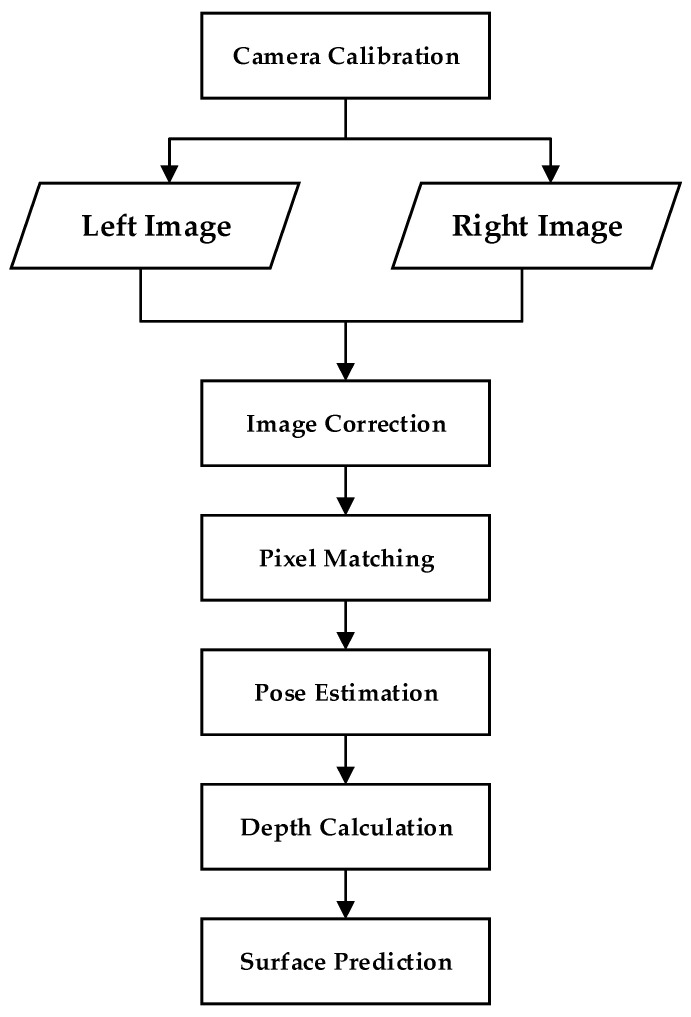
3D reconstruction process based on binocular vision.

The stereo vision method utilizes binocular cameras to capture different viewing angles of the scene or object to be measured. It calculates the object’s depth by analyzing the parallax of matching feature points in the images. The process is shown in Figure 3. The parallax of the binocular camera corresponds one-to-one to depth. As the depth value increases, the parallax value decreases. In other words, for the same parallax range, the corresponding depth range is larger. Binocular vision is low-cost and suitable for short-range measurements, but it has high texture requirements. It is very important in fields such as robotics as it can extract information about the relative positions of 3D objects near autonomous systems.

In 1960, Bela Julesz invented the random dot stereogram [84]. Consumer-grade RGB-D cameras are being used more frequently because of their affordability and portability. Izadi et al. [9] first proposed the Truncated Signed Distance Function (TSDF) model representation in KinectFusion, which simplifies model updating and enables real-time dense reconstruction using consumer-level binocular cameras. TSDF is a function that can describe the distance of a point from the surface of an object. A threshold for the distance of the 3D reconstruction is set, based on the SDF, and normalized to limit or “truncate” the distance beyond the threshold. By using distance fields, the representation of 3D shapes can be simplified, reducing the amount of data that needs to be stored.

**Figure 4:**
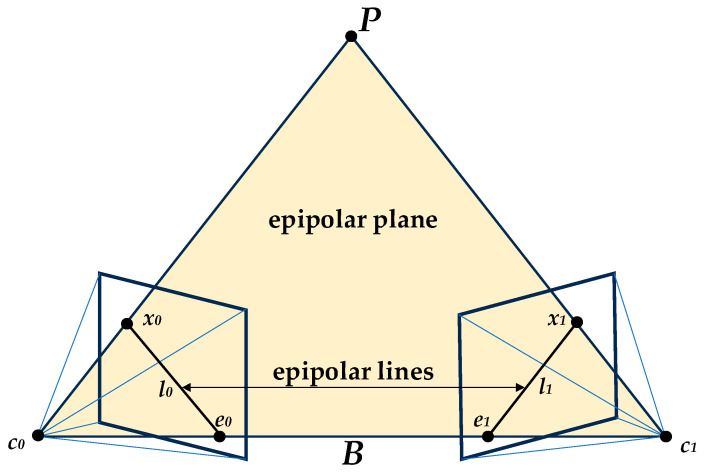
Epipolar geometric relations. It is acknowledged that the mapping point x_0_ in the left picture is on the epipolar line l_0_; then, the mapping point x_1_ in the right picture must be on the epipolar line l_1_.

In the binocular stereo vision system, epipolar geometry [85] describes a plane cluster with the binocular image baseline as the rotation axis. The object position *P* on a certain epipolar plane in this plane cluster is related to the optical centers of the left and right cameras of the binoculars. The geometric relationship that exists when c_0_ and c_1_ are coplanar is shown in Figure 4. In the binocular stereo vision system, the connection between the optical centers of the left and right cameras is the baseline B. B serves as the rotation axis of the epipolar geometric plane cluster. In the binocular stereo vision system, the connection between the optical centers c_0_ and c_1_ of the left and right cameras is the baseline B. The intersection lines of the epipolar plane and the image planes of the left and right binocular cameras are the left epipolar line l_0_ and the right epipolar line l_1_, respectively. The intersection points of the left and right epipolar lines with the baseline are the left pole points, respectively. e_0_ is the projection of the c_0_ onto the left pole e_1_, and e_1_ is the projection of c_1_ onto the right pole. The right epipolar line l_1_ and the imaging point x_1_ of the measured object on the right camera image plane are situated on the epipolar plane and the right camera image plane. Therefore, the image point x_1_ lies on the epipolar line l_1_. Thus, for the observed object *P* on the left camera, the matching point with the same name corresponding to the projection point x_0_ on the right image is constrained to l_1_, effectively reducing the search range for the corresponding point.

Whelan et al. integrated loop detection and loop optimization, utilized a deformation graph for non-rigid body transformation in real-time 3D rigid body reconstruction, and updated the coordinates of points based on the results of loop closure to align the two reconstructions [86], employing the surface element expression method [87]. Choi et al. combined numerous “model-to-model” local closed loops and larger-scale global closed loops to guarantee the global consistency of the reconstruction results. They divided the input RGB-D video stream into several scene segments as a group of frames and combined the geometric registration of the scene segments with global optimization [88]. Xin et al. [89] transferred the texture from the polarized surface to the fusion depth, utilized the depth map from the binocular camera to enhance the accuracy of the fusion depth, and applied the multiplier alternating direction method to optimize the reconstruction accuracy. In [90], the authors transferred the texture from the polarization surface to the fusion depth, used the depth map of the binocular camera to improve the accuracy of the fusion depth, and used the multiplier alternating direction method to optimize the reconstruction accuracy. Wang et al. [91] utilized calibration rods for calibration calculations based on epipolar correction and then used a weighted least squares filter to denoise and smooth the depth map, enabling the stable and accurate reconstruction of 3D point clouds in large scenes. Binocular cameras currently available on the market include ZED from Stereolabs in Paris, France, Kinect by Microsoft in Redmond, WA, USA, CamCube 3.0 by PMD in Siegen, Germany, Swiss Ranger 4000 by Mesa in Zurich, Switzerland, Bumblebee2Leap Motion by Point Gray in Vancouver, BC, Canada, Stereo IR 170 by Ultraleap in Bristol, UK, OAK camera by Luxonis Company in Mansfield, TX, USA, RealSense (D455) by Intel Corporation in Santa Clara, CA, USA, DUO by DUO3D Company in Henderson, NV, USA, etc.

#### 2.2.4. Structure from Motion (SFM)

In 1979, Ullman and Shimon proposed inferring the 3D structure and motion of objects through the two-dimensional transformation of the projected image [92]. In 1981, Longuet-Higgins and Tomasi proposed a method for recovering 3D structures from multiple images. This method is based on the relationship between camera motion and scene structure, utilizing the movement of the camera at different times or locations. Motion, which involves restoring the 3D structure of a scene through image sequences, is a crucial milestone in vision-based 3D reconstruction. SFM is mainly divided into four groups: incremental SFM [93], global SFM [94], hybrid SFM [95], and hierarchical SFM [96].

Judging from the input and output of the data stream, the SFM method takes a set of partially overlapping photos of the same object captured from various perspectives as input. The output includes the 3D reconstruction of the object and the internal and external parameters of the camera acquired during the reconstruction process. There are two main types of SFM: the factorization method and the multi-view geometry method.

**Figure 5:**
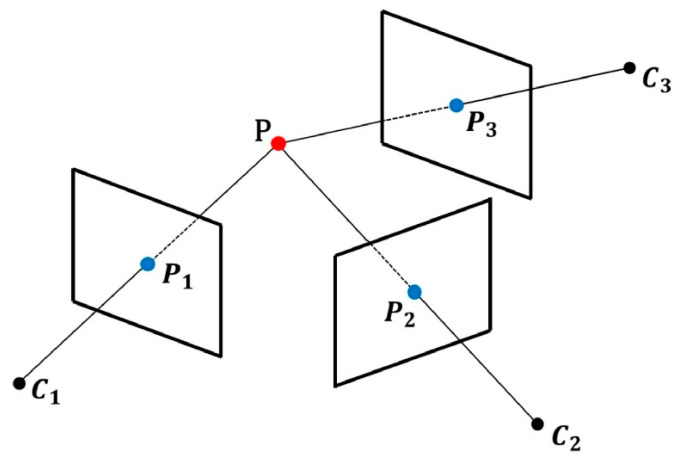
Multi-view geometric position.

(1) Factorization methods are mathematical models based on factorization, which obtain 3D structural information by decomposing image matrices [97]. Extract feature points from images captured at various viewing angles and then match them. These feature points can be corner points, edge points, and other points that have corresponding relationships in different viewing angles. The process involves converting the matched feature points into an observation matrix, which contains multiple feature point coordinates under each viewing angle. The next step is to factorize the observation matrix to decompose the factor matrix containing the 3D structure and camera motion information. Subsequently, the 3D structure information of the scene is extracted from the factor matrix, which includes the spatial coordinates of each feature point and the camera motion information, such as rotation and translation parameters, used to optimize the reconstruction results. Nonlinear optimization methods are typically utilized to enhance the accuracy of reconstruction. The advantage of the factorization method is that it can estimate the 3D structure and camera motion simultaneously without prior knowledge of the camera’s internal parameters. However, it is sensitive to noise and outliers, necessitating the use of suitable optimization methods to enhance robustness. Paul et al. [98] assumed that points are located on the object surface as a geometric prior to construct 3D point reconstruction and used affine and perspective cameras to estimate these quadratic surfaces and recover the 3D space in a closed form. Cin et al. [99] estimated the fundamental matrix by conducting motion segmentation on unstructured images to encode rigid motion in the scene. The depth map is used to resolve scale ambiguity, and the multi-body plane scanning algorithm is employed to integrate the multi-view depth and camera pose estimation network for generating the depth map.
(2) Multi-view 3D reconstruction is a method based on observing the same scene from multiple perspectives or cameras and reconstructing the 3D structure of the scene using image or video information [100], as illustrated in Figure 5. The MVS method has high requirements for image quality and viewing angle. It needs to address challenges such as inadequate viewing angle overlap and shadows. At the same time, the accuracy of the image matching algorithm also greatly impacts the reconstruction effect. The primary objective of image registration is to address variations in viewing angles and postures in scale and time, ensuring consistent geometric information. This, in turn, enhances the reliability and accuracy of the subsequent 3D reconstruction process. This method has high requirements for camera calibration.

Moulon et al. proposed a global fusion relative motion method [101] to achieve robust, accurate, and scalable 3D structure reconstruction. They used a camera pose estimation method based on SIFT features to calculate the relative motion between multiple perspectives. Motion relationships: Through a global optimization algorithm, this relative motion information is fused to achieve more accurate, robust, and scalable 3D structure reconstruction. Plan3d [102] efficiently handles occlusion within a restricted range by maximizing information from sparsely sampled viewpoints and hierarchically representing volumes. Zhu et al. [103] performed feature matching between synthetic images and ground images by employing descriptor search and geometrically constrained outlier removal, used synthetic depth and normal images to formulate oriented 3D patches, and combined the corresponding patches through patch-based matching. Relationships propagate to the bird’s-eye view.

The multi-view stereo vision algorithm first establishes correspondences between multiple views through feature point matching. It then utilizes basic matrix estimation techniques to calculate the relative posture between each view, followed by triangulation technology to merge the two-dimensional images from multiple views. The image coordinates are converted into a 3D point cloud. Finally, a dense depth map is obtained through a dense matching algorithm to ensure consistency in pixel brightness, adjacent pixel depth, and view visibility [104,105,106,107,108].

### 2.3. Introduction to 3D Reconstruction Technology

#### 2.3.1. Camera Calibration

Camera parameters are represented by a projection matrix, known as the camera matrix. The external parameters define the camera pose (position and orientation), while the internal parameters specify the camera image format (focal length, pixel size, and image origin). This process is often referred to as geometric camera calibration or simply camera calibration. Classic camera calibration requires specific features in the scene for locating and measuring, such as checkerboards or landmarks, while automatic camera calibration does not require them. They may leverage other means, such as automatically calibrating the camera by leveraging sensor data within the camera or by analyzing the geometry and structure of the image. There are many different approaches to calculate the intrinsic and extrinsic parameters for a specific camera setup. The most common methods include the Direct Linear Transformation (DLT) method, Zhang’s method [109], Tsai’s method [110], Calibration pole [111], and Selby’s method (specifically for X-ray cameras) [112]. Compared to traditional camera calibration methods, automatic calibration does not require any special calibration objects in the scene. In the visual effects industry, automatic camera calibration is often a component of the “match move” process. This process deals with synthetic camera trajectories and intrinsic projection models to re-project synthetic content into video [113].

#### 2.3.2. Image Local Feature Point Detection

Feature detection involves methods for computing abstractions of image information and making local decisions at each image point to determine whether a specific type of image feature is present at that point [114,115,116,117,118,119]. Features are subsets of the image domain, often considered in the form of points, continuous curves, or connected areas.

The method of blob detection (BLOB) mainly includes the methods of using the Laplacian of Gaussian operator [120], and the method employing the pixel Hessian matrix [121] (second-order differential) and its determinant value [122].

In 2004, Lowe proposed an efficient method, known as Scale-Invariant Feature Transform (SIFT) [123], which utilizes the convolution of the original image and a Gaussian kernel to establish the scale space. It extracts scale-invariant features on the Gaussian difference space pyramid. This algorithm exhibits affine invariance, perspective invariance, rotation invariance, and illumination invariance, making it the most widely used for enhancing image features.

The Speeded-up Robust Features (SURF) method is an enhancement of SIFT and enables quicker feature extraction through the utilization of integral images and rapid Hessian matrix detection. SURF also exhibits scale invariance and rotation invariance [124].

Corner point detection. Corner detection includes the Harris algorithm [125,126] and the FAST algorithm [127]. The Harris corner detector identifies corners by calculating the grayscale change in the local area of each pixel in the image. It utilizes the first-order and second-order derivative information of the grayscale image to identify corner points by computing a specific matrix. FAST is a high-speed corner detector that defines a circular area around a pixel and detects whether there are enough pixels that are brighter or darker than the central pixel to determine if it is a corner point.

Binary string feature descriptor. The BRIEF algorithm selects multiple pixel point pairs in the vicinity of the feature point, compares the gray values of these point pairs, and aggregates the comparison results into a binary string to represent the feature point. Finally, the Hamming distance is used to calculate whether the feature descriptors match [128]. The BRISK algorithm does not use FAST feature point detection in the feature point detection part but uses the more stable AGAST algorithm. In the construction of the feature descriptor, the BRISK algorithm uses simple pixel gray value comparison to obtain a cascade binary bit string to describe each feature point. BRISK adopts the neighborhood sampling mode, taking the feature point as the center of the circle, constructing multiple discretized Bresenham concentric circles with different radii, and then obtaining the same spacing on each concentric circle N sampling point [129]. The ORB algorithm uses FAST to detect feature points and then uses BRIEF to describe the feature points. It introduces a directional calculation method based on BRIEF and utilizes a greedy search algorithm to select point pairs, focusing on highly differentiated ones. Point pairs are used to represent binary strings [130]. Fast Retina Keypoint (FREAK) is a descriptor method that emphasizes speed and computational efficiency [131]. It generates efficient binary string descriptors by extracting features from the Retina model around key points and utilizing a rapid feature generation method.

#### 2.3.3. Image Segmentation

Image segmentation plays an important role in 3D reconstruction. It can help segment objects or scenes in the image into different areas, providing more accurate and meaningful information for subsequent 3D reconstruction. Image segmentation plays an important role in 3D reconstruction. Applications include object segmentation [132,133], background removal [134,135], contour extraction [136,137,138,139], semantic segmentation [140,141,142], dynamic scene segmentation [143,144,145,146,147], etc. Through effective image segmentation, the accuracy and stability of 3D reconstruction can be improved, providing a 3D model with more semantic information.

Edge-based methods, such as Canny edge detection and the Sobel operator, are utilized to detect object edges in images [148]. The region growing method is employed for segmenting images, point clouds, or voxel data. It does not require pre-specifying the number of segmentations and can handle areas of various shapes and sizes. Segments are formed by merging adjacent pixels with similar attributes. The optical flow method utilizes optical flow information between adjacent frames in the image sequence to achieve segmentation of dynamic objects [149,150]. The K-means algorithm is an iterative technique that divides the dataset into K clusters and assigns similar pixels to the same cluster to achieve image segmentation [151]. Deep Convolutional Neural Networks (DCNNs) are primarily utilized for pixel-level segmentation tasks in image segmentation [152,153]. Semantic segmentation networks are utilized to semantically annotate pixels in images, providing segmentation results with richer semantic information, including DeepLab [154] and PSPNet [155], among others. Instance segmentation networks like Mask R-CNN [156] are used to segment distinct instances in an image, particularly effective for scenes with multiple targets. Attention mechanisms have been introduced in image segmentation, such as Non-local Neural Networks (NLNet) [157], to enhance focus on crucial areas within the image. On April 5, 2023, Meta launched the Segment Anything semantic segmentation model [158]. The model’s generalization ability is considered a groundbreaking advancement in the field of computer vision (CV). It essentially addresses the generalization issue in deep learning for computer vision. Its pre-training model is suitable for various subjects. Scenes, objects, etc., that have not been trained have good segmentation capabilities.

Image segmentation algorithms can be selected based on specific scenarios and requirements. Traditional methods still perform well in some scenarios, while deep learning methods can typically deliver more accurate segmentation results when trained on large-scale datasets. Choose the appropriate one. The algorithm typically depends on the specific requirements of the application, computing resources, and data availability.

#### 2.3.4. Rendering

In 3D reconstruction, rendering is the process of projecting a 3D model onto a 2D image or display screen. It also serves the function of visualizing implicit surfaces. The rendering method plays a key role in 3D reconstruction and affects the final result of the reconstruction. The following are some common rendering methods used in 3D reconstruction:

(1) Rasterization rendering is a pixel-based rendering method that fragments the triangles of the 3D model into two-dimensional pixels and then colors each pixel, such as scanline rendering [159]. It has good real-time performance, but it struggles with handling transparency and reflection. It may not be as accurate as other methods when dealing with complex effects.
(2) Ray tracing rendering is a method of simulating the propagation of light in a scene. It calculates the lighting and shadows in the scene by tracing the path of the light and considering the interaction between the light and the object. It takes into account the reflection, refraction, shadows, etc., of the light [160]. Ray tracing produces high-quality images but is computationally expensive. Monte Carlo rendering estimates the rendering equation through random sampling [161] and uses Monte Carlo integration to simulate real lighting effects [162]. In order to improve rendering efficiency, Monte Carlo rendering uses Importance Sampling to select the direction of the light path.
(3) The radiometric algorithm is used to simulate the global illumination effect in the scene [163]. It considers the mutual radiation between objects and achieves realistic lighting effects by iteratively calculating the radiometric value of the surface.
(4) Shadow rendering is a technology that generates shadows in real time. It renders the scene from the perspective of the light source, stores the depth information in the shadow map, and then uses the shadow map in regular rendering to determine whether the object is in shadow, simulating the interaction between light and objects. The occlusion relationship between them is used to produce realistic shadow effects [164]. Shadow rendering is divided into hard shadows and soft shadows. In the former, there are obvious shadow boundaries between objects, while in the latter, the shadow boundaries are gradually blurred, producing a more natural effect.
(5) Ambient occlusion is a local lighting effect that considers the occlusion relationship between objects in the scene. It enhances shadows in deep recesses on the surface of objects, thereby enhancing the realism of the image [165].
(6) The non-photorealistic rendering (NPR) method aims to imitate painting styles and produce non-realistic images, such as cartoon style and brush effects [166].
(7) Volume rendering is a rendering technology used for visualizing volume data. It represents volume data as 3D textures and utilizes methods such as ray tracing to visualize the structure and features within the volume. The direct volume renderer [167] maps each sample value to opacity and color. The volume ray casting technique can be derived directly from the rendering equation. Volume ray casting is classified as an image-based volume rendering technique because the calculations are based on the output image rather than input volumetric data as in object-based techniques. The shear distortion method of volume rendering was developed by Cameron and Undrill and popularized by Philippe Lacroute and Marc Levoy [168]. Texture-based volume rendering utilizes texture mapping to apply images or textures to geometric objects.
(8) The splash operation blurs or diffuses the point cloud data into the surrounding area, transferring the color and intensity information of the points during the splashing process. This can be achieved by transferring the attributes of the point (such as color, normal vector, etc.) to the surrounding area using a specific weighting method. In adjacent splash areas, there may be overlapping parts where color and intensity superposition operations are performed to obtain the final rendering result [169].

The technology utilizes prior knowledge or models to improve the 3D reconstruction effect. These prior pieces of information can include the shape of the object, surface material, motion model, etc. [170]. By incorporating this information, the system can become more robust in handling challenges like noise and occlusion, enhancing accuracy and resilience to specific scenes or objects. Motion capture focuses on capturing and analyzing the movement of objects to provide precise data for 3D reconstruction and enhance the accuracy of the process.

## 3. Dynamic 3D Reconstruction Methods

Dynamic 3D reconstruction aims to capture and present the 3D structure of objects and environments, as well as their changes in dynamic scenes. It involves effectively handling dynamic factors such as moving objects, lighting changes, and scene evolution to create accurate and up-to-date images that reflect the current state of the scene. The essence of dynamic 3D reconstruction lies in capturing and modeling the 3D structure of an object or scene as it experiences dynamic changes, such as object movement, variations in lighting conditions, or environmental changes. Dynamic 3D reconstruction methods are typically based on techniques like feature point matching and motion estimation. Feature point matching is used to track key feature points in the scene, while motion estimation is used to estimate camera motion between adjacent frames.

### 3.1. Introduction to Multi-View Dynamic 3D Reconstruction

Multi-view dynamic 3D reconstruction involves utilizing multiple cameras or video cameras to observe the same scene from various perspectives and integrating temporal information to reconstruct the 3D structure of the dynamic scene. Observe the same scene from various angles, ensuring that all cameras can capture images simultaneously. Make sure that images taken by different cameras have consistent timestamps and that the matching and reconstruction results between adjacent frames are coherent. For each frame of an image, computer vision technology is used to extract feature points or feature descriptors in the image. By matching these feature points, the correspondence between different images is established. This process combines the pose information of the camera and the structural information of the scene. Simultaneously, scene modeling and camera positioning are carried out [171]. Dynamic scenes are processed through motion estimation, motion removal, and other technologies. The obtained 3D point cloud or model is then optimized and post-processed to enhance accuracy, remove noise, etc. [172,173].

Dynamic 3D reconstruction is primarily used for estimating the posture of the human body. By analyzing the captured data, the posture information of the human body at each time point is determined, including joint angles, body proportions, etc. Compared to general flexible body movements, human body movements have stronger priors. The shape of the human body conforms to a fixed geometric distribution. The SMPL/X model [174] or expanded versions of hands, faces, and other body parts are commonly utilized in academic circles [175] to describe the geometry of the human body using these parametric models. To achieve dense 3D reconstruction of multi-camera dynamic scenes, Matsuyama et al. proposed a parallel pipeline processing method [176] for reconstructing dynamic 3D object shapes from multi-view video images. Through this method, the time series of the full 3D voxel representation of the object’s behavior can be obtained in real-time, and the 3D object can be generated. 

### 3.2. Dynamic 3D Reconstruction Based on RGB-D Camera

In dynamic 3D reconstruction based on RGB-D cameras, depth information and color image data are input. Advanced computer vision algorithms and technologies are utilized to process data gathered by sensors to fulfill requirements such as real-time performance, reconstruction accuracy, and perception of dynamic objects. Dynamic 3D reconstruction algorithms based on binocular cameras generally involve processes such as identifying and tracking objects, estimating camera poses, calculating depth information, and creating 3D models in real time.

In 2016, Newcombe et al. proposed a real-time dynamic 3D reconstruction and tracking method for non-rigid scenes. DynamicFusion [177], a reconstruction algorithm that is not reliant on any template prior information, can be considered the pioneer of real-time dynamic reconstruction. The DynamicFusion system reconstructs the geometry of the scene while also estimating the 6D deformation domain of the dense volume representation, warping the estimated geometry into real-time frames. As more measurements are combined, a progressively denoised, detail-preserving, and more complete image is obtained. This method is suitable for a wide range of moving objects and scenes. However, DynamicFusion does not utilize any prior information, making the algorithm less robust to significant movements between frames and motions in occluded areas. It is more adept at handling closed topology. Surface reconstruction, especially the reconstruction of topological changes, is poor. Innmann proposed the Volume Deform algorithm [178], which combines global sparse color features (such as SIFT operators) and dense depth maps to enhance the robustness of finding accurate feature matching points, thereby significantly reducing the cumulative error of the reconstruction model. The shortcoming of this algorithm is the drift phenomenon. Although matching the global SIFT feature operator enhances the system’s robustness and reduces alignment errors, the drift phenomenon cannot be completely eliminated. Moreover, due to the regularization term setting, reconstructions of highly curved objects may appear smoother and less curved during motion.

Double Fusion [179], proposed by Yu et al., fully integrates the digitally driven template (SMPL model) with real-time reconstructed dense geometry, non-rigid motion, and inner human body shape. It introduces a double-layer surface representation: the inner layer, which is the parameterized model surface (inner body), and the outer surface obtained through deep fusion. Joint motion tracking based on a double-layer surface representation is proposed to ensure the robustness of the entire system, even during rapid motion. The shortcoming of the system is that when the user wears relatively thick clothing, the estimated human body also appears larger. Additionally, it cannot accurately distinguish between the outer surface and the interactions between people and objects.

Fusion4D [180] was proposed by Dou et al. The method is based on a multi-view scheme and does not rely on any prior information, allowing for the reconstruction of any scene or object in theory. As shown in the video, in addition to dynamically reconstructing the human body, it can also dynamically reconstruct dogs. An important contribution of this algorithm is the introduction of key volume, making it highly robust to large inter-frame motions and changes in mesh topology. Additionally, Fusion4D also incorporates voxel collision detection to ensure the correct TSDF model. The disadvantage of this system is that when the frame rate of the RGBD input stream is too low or the inter-frame motion is too large, the corresponding matching point estimation between frames will be inaccurate, leading to the failure of the non-rigid alignment process to converge.

Lin et al. proposed OcclusionFusion [181], which infers the motion of the occlusion area through Long Short-Term Memory (LSTM) and Graph Neural Network to calculate the confidence of the motion. This is achieved by modeling the network output using a probabilistic model confidence, thereby reducing implausible motion, enhances robust tracking, and ultimately improves reconstruction results. As a result, this method leads to improved results. Pan et al. [182] used an optimized epipolar geometric model and Mask R-CNN to jointly segment the image. They employed kernel principal component analysis to reduce point cloud noise and then applied an octree-based dynamic filtering method to eliminate outliers, ultimately achieving high-precision 3D reconstruction.

### 3.3. 3D Gaussian Splatting (3DGS)

**Figure 6:**
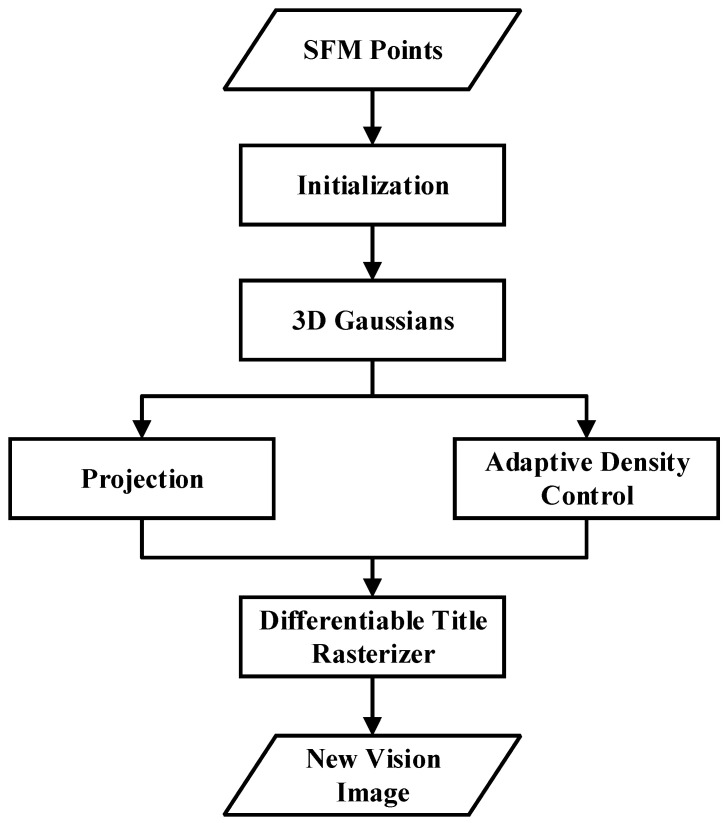
3D Gaussian Splatting system process.

Kerbl et al. proposed 3DGS, which utilizes 3D Gaussian functions to represent the scene. This method retains the characteristics of a continuous volume radiation field and introduces 3D Gaussian interleaving optimization, density control, and a fast visibility-aware rendering algorithm that supports anisotropic splattering. It ensures a real-time display rate while enhancing visual quality [183]. 3DGS maps point cloud data to the image plane and utilizes Gaussian functions to produce realistic images. Colors at different angles are represented using spherical harmonics to simulate the effect of viewing the scene from a different perspective. Spherical harmonics can attenuate high frequencies to a certain extent. Information is essentially a form of lossy compression that can convert discrete information into continuous information for computation [184]. The process of 3DGS is shown in Figure 6.

Antoine et al. [185] utilized Poisson reconstruction to extract meshes from Gaussian distributions, flatten the Gaussian sphere, bind Gaussian functions to the mesh surface, and jointly optimize these Gaussian functions and meshes through Gaussian splash rendering Physically based Newtonian dynamics can be seamlessly integrated into 3D Gaussian to achieve high-quality novel motion synthesis [185,186,187,188]. Chung et al. introduced a deep regularization method to avoid overfitting in few-shot image synthesis [189]. Geometric constraints are introduced by utilizing sparse and dense depth maps obtained from COLMAP and monocular depth estimation models, respectively. In order to prevent overfitting, this method incorporates unsupervised constraints on geometric smoothness and utilizes Canny edge detector to avoid regularization of edge regions with significant depth changes. The 4D Gaussian distribution incorporates a temporal component to model complex motion while maintaining efficiency [190,191]. Lin et al. proposed a progressive partitioning strategy called VastGaussian [192] based on 3D Gaussian distribution. This method divides a large scene into multiple units, optimizes these units in parallel, and then merges them into a complete scene. At the same time, decoupled appearance modeling is introduced into the optimization process to minimize appearance changes in rendered images, enabling high-quality reconstruction and real-time rendering of large scenes. Jiang et al. [193] combined the adaptive canonical point upsampling strategy and adaptive deformation to propose 3D Point Splatting Hand Reconstruction (3D-PSHR) to achieve real-time dynamic reconstruction of the pose-free hand. 3D-PSHR separates the appearance color into texture modeling with intrinsic albedo and pose-aware shading based on normal deformation. Chen et al. [194] introduced a unified representation model called Periodic Vibrating Gaussian (PVG). PVG extends the 3D Gaussian splatter paradigm to solve the problem of modeling large-scale scenes with complex geometries and unconstrained dynamics without relying on manually labeled object bounding boxes or expensive optical flow estimation. Gao et al. [195] combined grid representation with 3D Gaussian. By adopting Gaussian representation, not only the vertex positions but also the deformation gradients were used to guide the 3DGS. By utilizing the grid deformation method, this approach ensures real-time rendering and effectively maintains a high-quality appearance even when subjected to significant deformations.

Currently a very hot technology, 3DGS has revolutionary significance, redefining the boundaries of scene representation and rendering. It is expected to have a significant impact on the future progress of 3D reconstruction and representation.

### 3.4. Simultaneous Localization and Mapping (SLAM)

SLAM is primarily utilized to construct or update maps of unfamiliar environments while simultaneously tracking the location of targets within them. SLAM always utilizes multiple types of sensors, and various sensor types will result in different SLAM algorithms [196]. The SLAM algorithm utilizes visual and inertial sensors for data fusion to enhance the accuracy of attitude and motion estimation in dynamic scenes. Inertial information provides an important supplement to understanding the movement of objects that cannot be observed over a short period [197,198,199]. SLAM is primarily utilized to describe the mapping process employed when navigating in an unfamiliar environment. The SLAM system can run in real time (online SLAM) or process the collected data afterward (offline SLAM). In a dynamic environment, the system needs to process revisiting previous positions. Loop closure detection is a critical step in identifying and correcting errors that may accumulate during the mapping process. It involves using each new estimate to create an updated map during an iterative process [200,201,202]. Yan et al. [203] proposed GS-SLAM to integrate a 3D Gaussian representation into the SLAM system. GS-SLAM utilizes a real-time differentiable splatting rendering pipeline to greatly improve map optimization and RGB-D re-rendering speeds. GS-SLAM introduces an extended 3D Gaussian adaptive strategy designed to efficiently reconstruct newly observed scene geometries. Matsuki et al. [204] introduced a real-time SLAM system that utilized 3D-GS for incremental 3D reconstruction and introduced geometric verification and regularization to address ambiguities in incremental 3D dense reconstruction. This method is applicable to mobile single-lens cameras and RGB-D cameras.

Compared to static 3D scene reconstruction, dynamic 3D reconstruction involves changes in scene form such as moving objects, changing lighting, and evolving structures. These changes necessitate the use of various technologies in the field, including comprehensive motion estimation, recognition, and analysis. In the context of the Metaverse and General Artificial Intelligence (AGI), the increasing demand for real-time, high-precision, and intricate 3D scene reconstruction in complex environments is revealing a gap between current dynamic 3D reconstruction technology and application requirements.

## 4. 3D Reconstruction Methods Based on Machine Learning

### 4.1. Statistical Learning Methods

Statistical learning: Statistical learning methods can be utilized in 3D reconstruction to model and learn the mapping relationship from input data (such as images and point clouds) to 3D structures, learn scene and object shapes from large-scale data, and predict the 3D shape of objects through training models. This process enables the restoration and comprehension of 3D scenes [205,206,207].

### 4.2. 3D Semantic Occupancy Prediction Methods

3D semantic occupancy prediction methods utilize machine learning technology for semantic segmentation and scene understanding. This enables the improved identification and reconstruction of the geometric structure and semantic information of various objects in the scene.

Huang et al. utilized a TPV encoder (TPVFormer) [208] to efficiently extract TPV features and employed an attention mechanism to combine the image features related to each query in every TPV plane. A model trained solely with sparse point supervision can efficiently predict the semantic occupancy of all voxels. Ming et al. proposed a novel method based on a projection matrix for constructing local 3D feature volumes and global Bird’s Eye View (BEV) features. A global–local fusion module has been proposed to combine global information with local information to obtain the final 3D volume [209]. Li et al. [210] represented objects as a collection of deformable parts, enhancing the semantic consistency between the reconstructed mesh and the original image, and achieved single-view reconstruction through unsupervised learning.

### 4.3. Deep Learning Methods

Deep learning methods outperform most existing machine learning methods in several areas, with computer vision being a prominent one. With the advancement of deep learning technology, dynamic 3D scene reconstruction methods based on neural networks have started to capture the interest of researchers. Neural networks can discover feature information that humans may not be able to interpret, and it can extract high-dimensional features [211,212,213].

#### 4.3.1. Depth Map

Dou et al. proposed a technology based on deep neural networks (DNNs) to reconstruct a 3D face from a single 2D image in an end-to-end manner [214]. In 2018, Yao et al. proposed an end-to-end deep learning architecture called MVSNet for inferring depth maps from multi-view images. The method involves extracting depth visual image features initially and then constructing a 3D cost volume based on the reference camera frustum through differentiable monotonic distortions. Subsequently, 3D convolution is applied to regularize and regress the initial depth map. Finally, the reference image is utilized to optimize and generate the final output [215]. After MVSNet was proposed, it achieved very good results in estimating depth maps [216]. Sun et al. utilized a multi-scale approach to predict TSDF values, aiming to achieve higher-quality reconstruction accuracy. In addition, to address the issue of traditional 3D convolution consuming significant video memory, the 3D sparse convolution method is introduced to enhance operator efficiency. This method utilizes lower memory resources in exchange for higher-quality scene reconstruction and incorporates the 3D GRU module. To replace the traditional TSDF fusion method, consider using the GRU module, which can self-learn to enhance the model’s generalizability [217].

Objects in the real world almost never exhibit Lambertian reflection characteristics [218]. In 2017, DPSN [219] was used for the first time in the method of photometric stereo for three-dimensional reconstruction in response to the nonlinear relationship caused by non-Lambertian surface reflectance. On this basis, the calibrated photometric stereo method using orthogonal cameras and directional light sources is combined with deep learning, WJ20 [220], utilizing additional information, PS-FCN [221] employing supervised methods, GR-PSN [222], CNN-PS [223], NormAttention-PSN [224], DR-PSN [225], etc. Ikehata proposed a scalable universal photometric stereo network (SDM-UniPS) [226] that can operate reliably under unknown and arbitrary lighting conditions.

#### 4.3.2. Point Cloud

3D point cloud processing algorithms based on deep learning generally include voxel-based algorithms [227,228], view-based algorithms [229,230], and point-based algorithms [231,232]. The point-based algorithm directly uses point coordinates as input and can learn directly from the original data in an end-to-end manner, simplifying feature engineering and rule design in the traditional process. It has strong generalization ability and robustness and is suitable for scenarios of all types and sizes. Chen et al. proposed Point-BLS [233], which extracts point cloud features through a deep learning-based feature extraction network and then utilizes a comprehensive learning system for classification. Zhou et al. [234] used an instance segmentation method to extract and associate multiple key points on multi-view ISAR images and used an enhanced factorization method to derive the projection vector between the 3D geometry of the space target and the multi-view ISAR image. The 3D geometry reconstruction problem is transformed into an unconstrained optimization problem, and the 3D model is obtained using the quantum behavioral particle swarm optimization (QPSO) method.

Point cloud-based unsupervised representation learning (URL), which aims to learn robust and general feature representations from unlabeled data, has been intensively studied recently. This approach involves generating point cloud objects during training to alleviate the laborious and time-consuming challenge of data annotation [235]. Methods based on point cloud generation include point cloud self-reconstruction [236], point cloud GAN [237,238], point cloud upsampling [239], and point cloud completion [240,241], depending on the specific pre-task utilized. Methods based on point cloud context utilize context similarity for learning. Sanghi et al. [242] proposed enhancing feature representation by maximizing the mutual information between 3D objects and their local parts. Spatial context structures can also be used for learning. Poursaeed et al. [243] proposed learning the location of key points by predicting the rotation angle of 3D objects. Chen et al. [244] proposed learning the spatial context of objects by segmenting the distorted parts of the shape and correcting them.

#### 4.3.3. Neural Radiance Field (NeRF)

**Figure 7:**
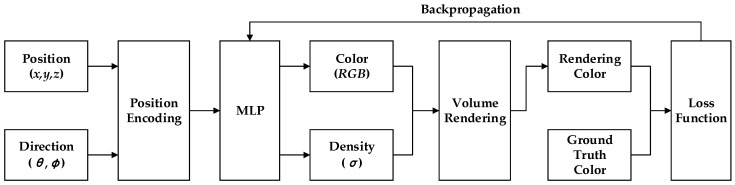
An overview of NeRF scene representation and differentiable rendering procedure.

Mildenhall et al. proposed a method called NeRF [245], which utilizes 5D neural radiation fields to represent complex geometry and material in continuous scenes. It is a new paradigm in the field of deep learning and computer vision, marking the transition from the conventional approach of deep learning to processing 3D data. NeRF utilizes the Multilayer Perceptron (MLP) network for parameterization and introduces a differentiable rendering method that enhances traditional voxel rendering techniques. RGB images are obtained through differentiable rendering. Each 5D coordinate is mapped to a higher-dimensional space using the position encoding method, enabling optimization of the neural radiation field to better express high-frequency details. Refer to Figure 7 for more details.

Barron et al. structurally replaced position encoding with integrated positional encoding and utilized multivariate Gaussians for approximation. By effectively rendering anti-aliased frustum cones instead of rays, the accuracy and efficiency of NeRF representation were significantly improved [246]. Wang et al. [247] introduced SDF as an implicit representation of 3D surfaces and proposed a volume rendering method based on SDF, enabling multi-view 3D reconstruction through volume rendering. The derivation of NeuS is result-oriented and directly constructs SDF. The relationship between weights and the sampling process also uses hierarchical sampling, similar to NeRF. Block-NeRF [248], proposed by Tancik et al., is used for perspective synthesis of large-scale scenes. By dividing the scene into blocks, the NeRF algorithm, which originally required a large number of calculations, is converted into calculations of small blocks, thus improving the scalability of the algorithm. Performance and operational efficiency: The Mega-NeRF [249] algorithm, proposed by Turki et al., introduces a new GPU-accelerated algorithm that can efficiently generate large-scale scenes with high-quality perspective synthesis. It offers better scalability and faster processing capabilities. Train the NeRF model, and by processing the input scene data in layers, you can effectively manage large-scale scenes and enhance the scalability and operational efficiency of the algorithm. The InstantNGP proposed by Müller is different from NeRF’s positional encoding. It uses a hash table to store features [250] and sets multiple resolutions to gather more information. This hash encoding idea can not only replace the positional encoding in NeRF but also be used for SDF network extraction, etc. NesF [251], proposed by Vora et al., provides a pre-trained NeRF model. It samples its volume density grid to obtain a 3D scene representation and converts the grid into semantics by utilizing a fully convolutional volume-to-volume network. A feature grid is used to obtain a geometrically reconstructed image. Mip-NeRF 360 [252] introduces a proposed MLP and distortion-based regularizer to achieve high-quality reconstruction. Geo-NeuS [253], proposed by Fu et al., explicitly performs multi-view geometry optimization by exploiting the sparse geometry of SFM and photometric consistency in multi-view stereo. Vinod et al. [254] trained a conditional NeRF without explicit 3D supervision by mapping input image pixels into texture space to learn 3D representations from a collection of single-view in-the-wild images of objects belonging to a specific category. Dai et al. [255] utilized scene context information and adopted a synthetic rendering formula to generate high-quality and harmonious 3D objects in existing NeRF. Li et al. [256] trained a 3D perception preprocessing network that integrates real-world degradation modeling to address the issue of information loss during image degradation and restoration by leveraging implicit multi-view guidance.

In graphics, the density of a 3D scene is an isotropic attribute. Unlike color, it has no viewing angle dependence. The output of NeRF is an image of the same scene captured from various viewing angles. Unlike explicit geometries, such as point clouds, voxels, and triangle meshes, that can be accessed by traversing all elements in the storage space, implicit geometry requires selecting spatial coordinates as input for sampling points. Neural implicit geometry involves converting the input and output through a neural network, enabling the rendering of pixel colors through weighted integration of a series of sampling points on the light source. The implicit scene will output the geometric density and color of these points [257]. By utilizing hierarchical sampling to address the issues of point waste and undersampling, NeRF can iteratively update parameters to refine the representation towards the actual value, enabling the completion of high-quality synthesis tasks from new perspectives. A NeRF neural network model can only store information about one object or scene, and NeRF is prone to overfitting for specific scenes [258,259].

In 3D reconstruction, deep learning is often combined with reinforcement learning methods [260,261,262,263]. The application of reinforcement learning in 3D reconstruction can help optimize data collection, path planning, reconstruction algorithms, and result optimization, thereby enhancing the efficiency, accuracy, and adaptability of three-dimensional reconstruction. The application of reinforcement learning, especially hierarchical reinforcement learning [264,265,266], in three-dimensional reconstruction can help robots better understand and perceive the environment [267], optimize the data collection process, improve reconstruction efficiency and quality, and adapt to different scenarios and environmental changes.

The loss function defines how to measure the difference between the current model output and the target output. The weight parameters needed for model learning are determined and adjusted by minimizing the results of the loss function. The commonly used error metrics include the mean absolute error (MAE) [268], mean square error (MSE) [269], normalized mean error (NME) [270], root mean square error (RMSE) [271], cross-entropy loss (CE) [272], adversarial loss [273], etc. Additionally, a customized loss function can be tailored to the specific requirements of tasks and models to more accurately align with the problem’s characteristics.

## 5. Datasets

In the study of 3D reconstruction, it is essential to consider that there may be variations in the 3D reconstruction outcomes across different scenarios. Therefore, when conducting experimental research, different datasets should be selected according to the specific research purposes. There are numerous datasets available for evaluating real-world and synthetic scene reconstruction methods. We collected and analyzed commonly used datasets for 3D reconstruction in Table 1, Table 2 and Table 3.

Many datasets contain both indoor and outdoor scenes, such as ETH3D [302], PASCAL3D+ [301], JRDB [314], etc. The dynamic 3D scene dataset is primarily utilized to assess the rendering quality of new perspectives in 3D scene reconstruction tasks. Given a captured video, the algorithm must accurately reconstruct the scene to generate images from a different perspective or time [315], commonly used datasets include Immersive Video [316], Neural 3D Video [317], Nerfies [318], Dynamic Replica [319], Bonn RGB-D Dynamic [320], etc.

## 6. Outlook and Challenges

### 6.1. Outlook

In indoor scenes, modeling the entire scene using a small number of color pictures remains the prevailing trend for the future. This is due to the limited availability of pictures in many indoor scenes, which restricts the opportunity to use a large number of images for training purposes. In outdoor scenes, it is also necessary to use multiple images for synthesis and training. At the same time, information from multiple perception modalities, such as images, laser scanning, depth sensors, and voice, can be integrated to enhance the accuracy of 3D reconstruction.

The application of hardware accelerators, such as GPUs, can significantly improve the calculation speed of 3D reconstruction algorithms. Cloud computing platforms can offer robust computing resources for large-scale data processing, supporting real-time performance and processing of extensive data requirements. The equipment used in the field of 3D reconstruction is gradually becoming simpler. The cameras and sensors on smartphones are powerful enough to support some simple 3D reconstruction applications. The popularity of mobile devices enables users to easily conduct image-based 3D scanning and 3D reconstruction [321]. 3D reconstruction software tools enable users to perform 3D modeling without requiring an in-depth understanding of complex algorithms and principles. This accessibility allows a growing number of individuals to utilize 3D reconstruction technology across various application fields, thereby advancing the development of this field.

In February 2024, OpenAI launched a new model, Sora, designed to generate videos based on text input. In March 2024, Figure AI integrated ChatGPT as an intelligent brain, which will be an important milestone in the development of AGI. The application of 3D reconstruction in the metaverse is accompanied by the development of AR/VR products by technology companies such as Apple, Meta, Google, and Sony. Some examples include Apple Vision Pro, Meta Quest 3, Microsoft HoloLens 2, VIVE Pro, and PlayStation VR 2. With the advancement of these products, individuals can work in the metaverse, engage in face-to-face work from home, immerse themselves in 3D scenes, and experience movies and games in an immersive manner. By combining robotics with brain–computer interface technology, people can interact with machines using their bodies to complete various tasks in work and life, truly liberating human hands.

### 6.2. Challenges

3D reconstruction is an open research field. Although vision-based 3D reconstruction has made remarkable progress, there are still some challenges. Challenges such as managing dynamic scenes, occlusions, topology changes, and efficiently processing large-scale data still necessitate further in-depth research. The extensive data collection involved in 3D reconstruction may raise privacy and ethical issues, particularly in public places and personal areas.

The accuracy of 3D reconstruction is affected by sensor noise, changes in shooting conditions, and environmental lighting, which result in data uncertainty. Many 3D reconstruction algorithms have limited robustness to various scenes, lighting conditions, and object types, which can lead to failures or performance degradation in specific scenarios. High-quality 3D reconstruction is computationally expensive, and finding ways to efficiently create a realistic scene model using readily available equipment is currently a significant challenge. Challenges are not only problems but also the source of innovation. By overcoming these challenges, 3D reconstruction will better serve the development of society and technology.

## 7. Summary

This survey analyzes the key technologies of 3D reconstruction from the aspects of static and dynamic scenes, machine learning, etc. It introduces the active vision method and passive vision in detail and summarizes the research progress of various 3D reconstruction methods and the effects of 3D reconstruction. Different application fields have varying requirements for 3D reconstruction, and distinct 3D scenes should be reconstructed to serve specific task-oriented purposes.

With the development of science and technology, artificial intelligence will eventually reach or even surpass human intelligence in the future. With the aging of the population in the future, robots will inevitably replace humans in various tasks. Vision-based 3D reconstruction will provide accurate visual perception information and help the model better transition from the two-dimensional world to the 3D world. Continuous innovation in this field will equip computers with more advanced tools and perspectives to comprehend and utilize the 3D world. This will enable us to understand and simulate the physical world better, thus enhancing the intelligence of robots.

## Figures and Tables

**Table 1:** Human body datasets.

Dataset	Persons	Total of Data	Type of Data
Human3.6M [274]	11	3.6 million	images
MPII-Pose [275]	/	25K	images
BUFF [276]	5	11,054	3D scans
UP-3D [277]	/	7126	images
SHPD [278]	/	23,334	images
SMPL-X [279]	31	5586	images, 3D scans
THUman [280]	230	7K	images
HUMBI [281]	772	67 million	images
HUMAN4D [282]	4	50,306	mRGBD, meshes
GRAB [283]	10	1.6M	images
MVP-Human [284]	400	6K, 48K	3D scans, images
3DPeople Dataset [285]	80	2.5 million	images

**Table 2:** Indoor scene datasets.

Dataset	Total of Data	Type of Data	Scenes	Objects
TUM RGB-D [286]	39 sequences	images, depth	39	/
NYUD2 [287]	1449	images, 3D point cloud	464	894
SUN 3D [288]	415 scenes	images, video	254	41
NYU v2 [289]	407,024	images, depth	464	894
ShapeNet [290]	300M	CAD	/	3135
SUNRGBD [291]	10,335	images	47	700
SceneNet RGB-D [292]	5M	images	57	255
SceneNN [293]	100 scenes	images, 3D meshes	100	/
SUNCG [294]	130,269	depth, 3D meshes	24	84
CoRBS [295]	20 sequences	images	20	20
Matterport3D [296]	194,400	images, 3D meshes	90	10,800
2D-3D-S [297]	70,496	images, 3D point cloud	11	13
Scannet [298]	2.5M, 36123	images, 3D point cloud	1513	21
InteriorNet [299]	20M, 1M	images, CAD	15k	/

**Table 3:** Outdoor scene datasets.

Dataset	Total of Data	Type of Data	Scenes	Objects
KITTI [300]	41K	images	22	80,256
PASCAL3D+ [301]	22,394	images, CAD	/	13,898
Eth3D [302]	24 megapixels	images, 3D point cloud	/	/
Semantic3D [303]	4 billion points	images, 3D point cloud	30	8 classes
Paris-Lille-3D [304]	57.79 million	images, 3D point cloud	2	50 classes
ApolloCar3D [305]	5277	images	/	60k
Cityscapes 3D [306]	5000	images, 3D point cloud	/	8 classes
BlendedMVS [307]	17k	images, 3D meshes	113	/
CSPC-Dataset [308]	68 million points	images, 3D point cloud	5	6 classes
Toronto-3D [309]	78.3 million points	images, 3D point cloud	/	8 classes
STPLS3D [310]	16 km^2^	images, 3D point cloud	/	/
KITTI-360 [311]	300k, 1 billon points	images, 3D point cloud	/	/
DiTer [312]	/	images, 3D point cloud	/	/
SubT-MRS [313]	30 scenes	images, 3D point cloud	30	/
